# Exploring the Regulatory Potential of Long Non-Coding RNA in Feed Efficiency of Indicine Cattle

**DOI:** 10.3390/genes11090997

**Published:** 2020-08-25

**Authors:** Pâmela A. Alexandre, Antonio Reverter, Roberta B. Berezin, Laercio R. Porto-Neto, Gabriela Ribeiro, Miguel H. A. Santana, José Bento S. Ferraz, Heidge Fukumasu

**Affiliations:** 1 Department of Veterinary Medicine, Faculty of Animal Science and Food Engineering, University of Sao Paulo, Pirassununga, Sao Paulo 13635-900, Brazil; roberta.berezin@gmail.com (R.B.B.); gabriela.ribeiro.br@gmail.com (G.R.); jbferraz@usp.br (J.B.S.F.); fukumasu@usp.br (H.F.); 2 Commonwealth Scientific and Industrial Research Organization, Agriculture & Food, St. Lucia, Brisbane, QLD 4067, Australia; Toni.Reverter-Gomez@csiro.au (A.R.); laercio.portoneto@csiro.au (L.R.P.-N.); 3 Department of Animal Science, Faculty of Animal Science and Food Engineering, University of Sao Paulo, Pirassununga, Sao Paulo 13635-900, Brazil; miguel-has@hotmail.com

**Keywords:** *Bos indicus*, co-expression network, residual feed intake, RNA-Seq

## Abstract

Long non-coding RNA (lncRNA) can regulate several aspects of gene expression, being associated with complex phenotypes in humans and livestock species. In taurine beef cattle, recent evidence points to the involvement of lncRNA in feed efficiency (FE), a proxy for increased productivity and sustainability. Here, we hypothesized specific regulatory roles of lncRNA in FE of indicine cattle. Using RNA-Seq data from the liver, muscle, hypothalamus, pituitary gland and adrenal gland from Nellore bulls with divergent FE, we submitted new transcripts to a series of filters to confidently predict lncRNA. Then, we identified lncRNA that were differentially expressed (DE) and/or key regulators of FE. Finally, we explored lncRNA genomic location and interactions with miRNA and mRNA to infer potential function. We were able to identify 126 relevant lncRNA for FE in *Bos indicus*, some with high homology to previously identified lncRNA in *Bos taurus* and some possible specific regulators of FE in indicine cattle. Moreover, lncRNA identified here were linked to previously described mechanisms related to FE in hypothalamus-pituitary-adrenal axis and are expected to help elucidate this complex phenotype. This study contributes to expanding the catalogue of lncRNA, particularly in indicine cattle, and identifies candidates for further studies in animal selection and management.

## 1. Introduction

The flow of information from DNA to protein synthesis comprises specific steps in which gene expression can be controlled. Long non-coding RNA (lncRNA) are thought to play a role in controlling gene expression, making this type of molecule particularly interesting in the context of complex phenotype regulation both in humans [1] and in livestock species [2]. Structurally, lncRNA have low or no potential for protein-coding and are lowly conserved among species [3]. They present no specific sequence pattern, making categorization of lncRNA and prediction of their function challenging [2]. Therefore, lncRNA are considered evolutionarily less conserved than protein-coding genes, which does not indicate a lack of function, but rather a possible fast adaptation mechanism [3]. These molecules can fold into complex structures, mediating target recognition not only by base pairing but also by tertiary structural interactions [3]. In contrast to protein-coding genes, lncRNA are more tissue-specific, are expressed at lower levels and often contain multiple exons, polyA tail, 5’ cap and CpG islands in their promoter regions [2]. These intriguing characteristics of lncRNA confer on these molecules the ability to perform several regulatory functions.

Among other functions, lncRNA are involved in recruiting chromatin-modifying complexes, regulating DNA methylation levels, modulating allele-specific expression and alternative splicing, and functioning as guides, precursors or sequester of miRNA, transcription factors or multiprotein complexes [4,5]. The variety of mechanisms of action exhibited by lncRNA make these molecules attractive candidates to investigate when developing strategies aimed at regulating important traits for animal production, such as feed efficiency. Feed efficiency (FE) contributes to both increased productivity and reduced environmental impact of livestock. Indeed, in the last few years, there is increasing evidence that lncRNA play in role in many physiological outcomes that impact on the productivity of bovines, such as lactation [6], mastitis [7,8], skeletal muscle development [9], heat stress [10], acidosis [11] and spermatogenesis [12].

For FE-related traits, such as residual feed intake (RFI), several studies have evaluated mRNA expression in relevant tissues in an attempt to uncover the molecular basis of observed phenotypes [13,14,15,16,17]. However, there is limited information available on the role of lncRNA in FE. In cattle, a catalogue of lncRNA in multiple tissues was published by [18] and there was a limited overlap of lncRNA described across studies in bovine [2], demonstrating the multi-specificity character of lncRNA including tissue, cell, time and condition specificity. This highlights the need to study these lncRNA molecules specifically in the context of FE.

Although the most common way of identifying relevant lncRNA is by differential expression (DE) between contrasting conditions, methodologies based on “guilty-by-association” such as clustering and correlation (co-expression networks) with known proteins provide more efficient means to predict the role of important lncRNA for specific phenotypes [19]. Recently, Nolte et al. [20] explored the regulatory role of lncRNA in metabolic efficiency of taurine crossbred beef cattle and were able to identify eight potential regulators with activity in muscle and liver, based on the bovine reference genome UMD3.1. This group also explored the regulatory potential of liver antisense lncRNA in FE using the same animals but using the new bovine reference genome (ARS-UCD1.2) [21]. Taurine and indicine breeds differ in their ability to perform in different environments [22,23,24], which can be partly attributed to their differing ability to utilize certain forages. As most studies on FE are performed using taurine breeds, and it is not clear if the mechanisms underlying FE differ between taurine and indicine sub-species, our study was aimed at identifying novel lncRNA in indicine beef cattle with the potential to regulate FE.

We chose to investigate lncRNA in liver and muscle based on their known role in FE [14,16,17]. We also chose to investigate tissues from the hypothalamus-pituitary-adrenal (HPA) axis, a major neuroendocrine system responsible for controlling stress response, circadian rhythm, hunger, energy storage/expenditure, sexual behavior, immune system function and temperament. The dataset used in the current study was originally compiled to explore mRNA expression associated with FE. We previously reported an important role for genes encoding *NR2F6* and *TGFB1* in the regulation of hepatic inflammatory response and muscle tissue development, respectively [25]. We also identified other potential biomarkers of FE related to hormonal control of metabolism and sexual maturity. Nevertheless, we see RNA-Seq data as a rich source of information and hypothesize that additional investigation of the role of lncRNA in FE represents a critical next step in elucidating the molecular mechanisms involved in the regulation of this complex trait and the identification of additional key regulators of FE which could be explored in the context of both animal selection and animal management.

## 2. Materials and Methods

### 2.1. Data Acquisition

We used RNA-Seq data (Illumina HiSeq2500, 100 bp, paired-end) from a database comprised of 86 tissue samples from high and low FE Nellore (*Bos indicus*) bulls, including liver (nine high FE and nine low FE), skeletal muscle (eight high FE and nine low FE), adrenal gland (seven high FE and eight low FE), hypothalamus (nine high FE and nine low FE) and pituitary gland (nine high FE and nine low FE). Briefly, 98 Nellore bulls (16 to 20 months old and 376 ± 29 kg BW) were evaluated in a 70-d feeding trial. Total mixed ration was offered ad libitum, daily dry matter intake (DMI) was individually measured and animals were weighed every fortnight. RNA-Seq data was generated from tissues collected from high and low FE animals, selected as the extremes of residual feed intake (RFI) phenotypes (high FE = −1.44 ± 0.29 kg/day; low FE = 1.57 ± 0.46 kg/day; *p*-value = 2.0 × 10^−10^) [26]. The two groups also differ regarding DMI (high FE = 9.6 ± 1.57 kg/day; low FE = 12.23 ± 0.9 kg/day; *p*-value = 8.0 × 10^−4^), but not regarding average daily gain (high FE = 1.88 ± 0.55 kg; low FE = 1.76 ± 0.27 kg; *p*-value = 0.57). This RNA-Seq dataset is publicly available in the European Nucleotide Archive (ENA) as part of the Functional Annotation of Animal Genomes Consortium (FAANG) under the study ID PRJEB27337. Refer to Alexandre et al. [27] for details regarding the experimental design and characterization of animals into high and low FE groupings. For details about RNA libraries and previous results regarding mRNA expression in the tissues used here, refer to Alexandre et al. [25].

### 2.2. Identification of New Transcripts

**Figure 1:**
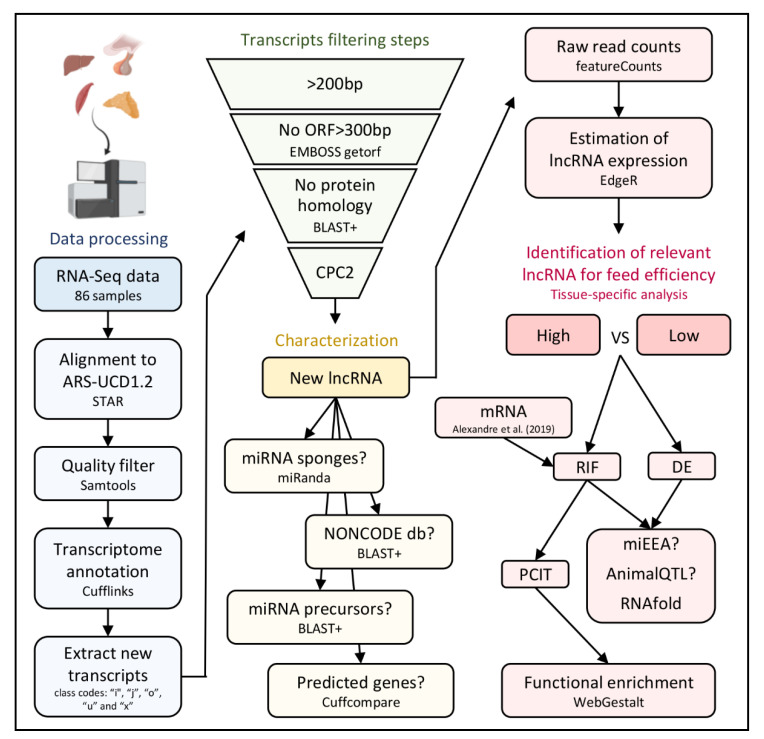
Pipeline used to identify and characterize new Long non-coding RNA (lncRNA) and identify relevant lncRNA for feed efficiency in Nellore cattle. DE—Differential expression; RIF—Regulatory Impact Factor; PCIT—Partial Correlation and Information Theory.

Libraries were constructed from polyA-tail selected transcripts and were aligned to the new bovine reference genome (ARS-UCD1.2) using STAR 2.2.1 [28]. Secondary alignments, duplicated reads and reads failing vendor quality checks were removed using Samtools [29]. Cufflinks software [30] was used to generate one annotation file for each sample using the annotation file from NCBI (GCF_002263795.1_ARS-UCD1.2_genomic.gtf) as a reference. Then, Cuffmerge was used to combine the individual annotation files and the reference into one single annotation that represents the combined transcriptome of the five tissues. In this annotation, the transcripts were classified according to their genomic position in relation to known genes and arbitrary names were assigned to the ones that were not a perfect match [31]. Therefore, it was possible to extract only those transcripts from class codes: “i” (intron transcripts), “j” (new isoforms), “o” (generic overlap with known exon), “u” (intergenic transcripts) and “x” (overlap with known gene on the opposite strand). Refer to Figure 1 for an overview of the analytical pipeline.

### 2.3. Identification of lncRNA

Once new transcripts with the potential to be lncRNA were selected, this annotation file together with the bovine reference genome (ARS-UCD1.2) was used to generate a fasta file containing the sequence of the new transcripts. New transcripts were then subjected to a four-step filtering procedure as follows: (1) Transcripts smaller than 200 bp were excluded since, by definition, only transcripts >200 bp are considered long non-coding RNA. (2) Then, the EMBOSS getorf tool [32] was used to exclude transcripts with ORFs greater than 300 bp between a START and a STOP codon. This threshold was determined based on the fact that most proteins recorded in eukaryotes have more than 100 amino acids [33]. (3) Next, the transcripts were tested for similarity with the UniProt/SwissProt database using the BLAST+ Blastx tool [34]. Transcripts with significant homology to the database (E-value < 10^−6^) were excluded. (4) The transcripts were finally tested for their coding potential using the online tool CPC2 [35]. Transcripts that passed through all four filters and presented more than one exon were considered new lncRNA.

### 2.4. General Classification of lncRNA

To characterize the new lncRNA, an annotation file containing only the new lncRNA was generated and compared, using Cuffcompare [36], with an NCBI annotation file containing both known genes and also genes predicted using a gene prediction method called GNOMON (http://www.ncbi.nlm.nih.gov/genome/guide/gnomon.shtml). The GNOMON method is based on the comparison of complete or partial sequences of proteins from model organisms. The identified lncRNA were also compared with previously described lncRNA in cattle, present in the NONCODE database [37] using the BLAST+ Blastn tool [34]. Homologies with E-value < 10^−6^ were considered significant.

### 2.5. lncRNA and miRNA

Among the various functions that lncRNA can perform, two involve miRNA: lncRNA can either be precursors of miRNA or act as “sponges,” attaching to miRNA and preventing them from performing their role in inhibiting mRNA expression. To identify lncRNA that could act as precursors of known miRNAs, bovine miRNA precursor sequences were downloaded from mirBase (http://www.mirbase.org/) and aligned to the identified lncRNA using the BLAST+ Blastn tool [34]. The lncRNA which matched the miRNA precursors with E-value < 10^−6^ were considered significant. To test whether the identified lncRNA could be targets of miRNA, the miRanda software was used [38] to identify miRNA binding sites in two stages. First, local alignment is made between each miRNA and each lncRNA generating a score based on complementarity. Then, the thermodynamic stability of high-score alignments (>160) is calculated using folding routines of the RNAlib library, which are part of the ViennaRNA package [39]. Finally, identified targets with energy below the −20 kcal/mol threshold are reported.

### 2.6. lncRNA Expression

The annotation file generated by Cuffmerge representing the combined transcriptome of the five tissues was used to extract the raw read counts per sample at transcript level using featureCounts [40]. Then, considering only the identified lncRNA and each tissue, the EdgeR package [41] was used to normalize the counts by trimmed mean of M-values (TMM). For each tissue, only lncRNA presenting at least 1 count per million (CPM) in at least half of the samples in that tissue were considered for further analysis.

Differential expression analysis was performed to identify lncRNA molecules DE across tissues using a *t*-test with *p* < 0.01. This method, as opposed to more stringent methods such as the one applied by EdgeR, was used because rather than identifying high confidence DE lncRNA we wanted to prioritize lncRNA for further exploration in co-expression analysis. This approach has been extensively applied in other studies [16,25,42] and is appropriate in the context of lncRNA expression, as we expect these molecules to have a regulatory role similar to transcription factor, meaning a small change in gene expression can have a significant influence on the expression of other mRNA [20,43]. The Venn diagram was produced using InteractVenn [44].

### 2.7. lncRNA and mRNA

To explore the relationship between lncRNA and mRNA, mRNA expression was also estimated using featureCounts [40] and the bovine annotation file from NCBI (GCF_002263795.1_ARS-UCD1.2_genomic.gtf) at the gene level. As for the lncRNA, the EdgeR R package [41] was used to normalize the counts by TMM for each tissue and only genes presenting at least 1 CPM in at least half of the samples were considered for the analysis.

The regulatory potential of lncRNA was tested using Regulatory Impact Factor metrics (RIF, [45]). This metric assigns scores to genes/transcripts that are consistently differentially co-expressed with target genes, and to those with the most altered ability to predict the abundance of target genes. This approach has been applied to several biological circumstances [46] and recently in the context of lncRNA regulation [20]. As target genes, we used the mRNA listed in our previous work [25] as relevant in the context of FE in beef cattle. Therefore, for each tissue, we created a specific set of target mRNA based on them being DE, tissue-specific (TS) or identified as key regulators (transcription factors). Moreover, we updated the list of genes considered in our previous work for harboring SNPs associated with FE, considering the AnimalQTL database—release 41 [47]. These genes were included in all tissue datasets. The lncRNA with RIF scores deviating ±2.57 SD from the mean (corresponding to a nominal *t*-test *p*-value of <0.01) were considered significant and labeled as key lncRNA.

As discussed previously, methodologies based on “guilty-by-association,” such as co-expression networks comprising both lncRNA and known proteins, can be used to predict the role of lncRNA in a specific biological context [19]. Therefore, for each tissue, we created a co-expression network containing all lncRNA and the relevant mRNA for FE (used in the RIF analysis), using the Partial Correlation and Information Theory (PCIT) algorithm [48].

### 2.8. Functional Analysis

The miRNA with binding sites on DE or key lncRNA were tested for functional enrichment using the MiEEA online platform [49] using Fisher’s exact test and Benjamini-Hochberg correction (*p* < 0.1). Also, the genomic position of DE and key lncRNA was used to assess whether they were located in regions of quantitative trait loci (QTL) described in the Animal QTLdb [47]. The structure of some lncRNA were determined using the minimum free-energy and partition function and avoiding isolated base pairs using the Vienna RNA package on the online platform RNAfold WebServer v.2.4.13 [50]. Finally, to gain insight into the function of the lncRNA, we performed a functional enrichment of KEGG pathways and Gene Ontology Biological Processes using their co-expressed mRNA in the online tool WebGestalt [51]. WebGestalt uses a hypergeometric test and Benjamini-Hochberg correction to test for enriched terms (FDR < 0.1) and when appropriate, enriched terms were grouped by similarity based on affinity propagation.

## 3. Results

### 3.1. New lncRNA

Using gene expression data from 86 samples of the hypothalamus, pituitary gland, liver, skeletal muscle and adrenal gland from Nellore cattle, it was possible to identify 132,117 new transcripts. Of these, 132,035 (99.9%) were longer than 200 bp, a criterion used to distinguish small from long ncRNA. The filter that excluded most transcripts was the requirement for ORFs to be greater than 300 nt, which resulted in only 6.6% (8709) of the transcripts initially identified being considered. Then, 3665 transcripts were excluded because they showed high similarity with known proteins from the UniProt database (E-value < 10^−6^) and seven were excluded because they had coding potential according to CPC2 analysis. Finally, 3087 novel transcripts passed through all filters and presented more than one exon. These were considered new lncRNA. The gtf file with the annotation of these new lncRNA can be found in Supplementary File S1.

### 3.2. Characteristics of New lncRNA

**Figure 2:**
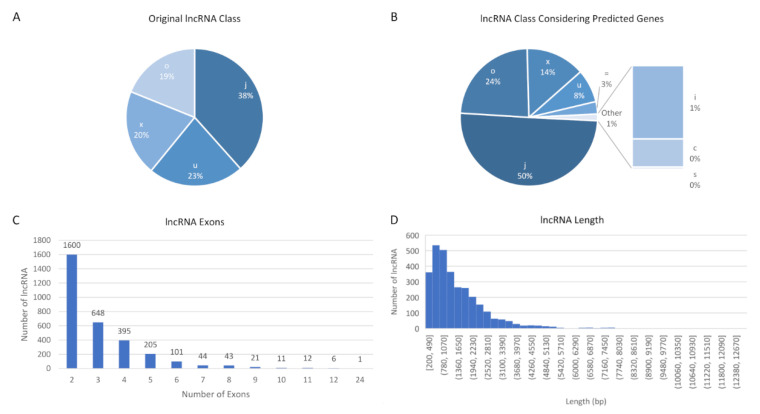
Classification of lncRNA. Class codes in relation to known genes (**A**) and known genes + algorithm-predicted genes (**B**); number of exons (**C**); and length in base pairs (**D**). Classification of lncRNA was according to Trapnell (2017), class codes are: = “-“ — Complete match of intron chain; “c”—Contained; “j”—Potentially novel isoform (fragment): At least one splice junction is shared with a reference transcript; “i”—A transfrag falling entirely within a reference intron; “o”—Generic exonic overlap with a reference transcript; “u”—Unknown, intergenic transcript; “x”—Exonic overlap with reference on the opposite strand; “s”—An intron of the transfrag overlaps a reference intron on the opposite strand (likely due to read mapping errors).

In the cufflinks pipeline, transcripts are classified regarding their position in relation to the nearest known genes [31]. Among the 3087 transcripts identified as novel lncRNA, the majority were in class code “j”—isoforms of known genes (Figure 2A). When we compared the lncRNA identified with the annotation containing genes predicted by gene search algorithms (Gnomon, NCBI), we found that even more transcripts (1550) were classified as class “j” (Figure 2B). A few transcripts (*n* = 90) overlapped perfectly with the predicted genes (class “=”). There was also a small number of lncRNA (*n* = 13) in classes “c” (contained in a known gene) and “s” (*n* = 1, overlap with reference intron on the opposite strand). From the 695 transcripts initially classified as intergenic (“u”), 392 actually overlapped with regions of predicted genes.

The number of exons per lncRNA generally varied between two (*n* = 1600) and 12 (*n* = 6), but one lncRNA presented 24 exons (Figure 2C). Regarding the length of the transcripts, 75% contained up to 2000 bp and the longest lncRNA contained 12,921 bp (Figure 2D). The lncRNA were evenly distributed between DNA strands, with 1541 in the negative-sense and 1546 in the positive-sense strand. Moreover, the 3078 lncRNA transcripts corresponded to 2330 loci, of which 448 presented multiple transcripts/isoforms, varying from two (251 loci) to 30 (1 locus). A complete table of attributes for each new lncRNA identified here can be found in Supplementary File S2.

Although often not included in the reference annotation, several studies have identified lncRNA expressed in cattle and other species. These results form the basis of databases such as NONCODE [37], which was used to verify whether the lncRNA found here had been previously described. Of the 3087 lncRNA identified, 1686 (55%) showed high similarity with 804 previously identified lncRNA in bovine (E-value < 10^−6^). Although in most cases one new lncRNA presented similarity with one previously described lncRNA, there were cases where multiple new lncRNA were significantly similar (E-value < 10^−6^) to the same lncRNA in the NONCODE database. The most extreme example was NONBTAT026662.2, which was significantly similar to 199 of our new lncRNA located in different chromosomes and with identity matches varying from 77.3% to 96.8%.

When we tested the similarity between our lncRNA and the database of miRNA precursors, only 45 lncRNA were identified as possible miRNA precursors (E-value < 10^−6^). A different result was obtained regarding the possible role of lncRNA as miRNA sponges, with 2944 lncRNA (95%) containing binding sites for up to 48 miRNA in a single transcript.

**Figure 3:**
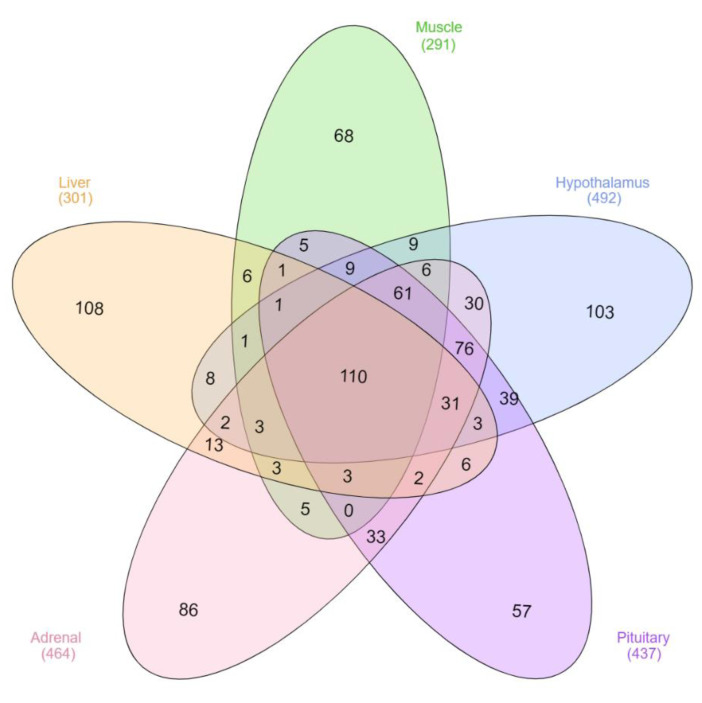
Number of expressed lncRNA identified per tissue type.

Of the total lncRNA identified, 464 were expressed above the minimum threshold in adrenal gland, 301 in the liver, 291 in muscle, 437 in pituitary gland and 492 in hypothalamus, and a total of 110 were expressed in all tissues (Figure 3). The hypothalamus, pituitary gland and adrenal gland had 76 lncRNA expressed in common. These three tissue types plus muscle had 61 lncRNA expressed in common. The pituitary gland expressed 39 lncRNA in common with hypothalamus and 33 in common with the adrenal gland.

### 3.3. Differentially Expressed lncRNA

Differential expression analyses identified 13 lncRNA differentially expressed in the adrenal gland, 10 in liver, 14 in muscle, nine in hypothalamus and 17 in the pituitary gland, relative to other tissues (Table 1, Supplementary File S3). Of those, three were DE in two or three tissues, resulting in a total of 59 unique DE lncRNA. TCONS_00222966 was DE in the hypothalamus and liver; TCONS_00223090 in the adrenal gland and pituitary gland; and TCONS_00141903 in the adrenal gland, hypothalamus and pituitary gland. The first two lncRNA, TCONS_00222966 and TCONS_00223090, are located in non-assembled portions of the genome, but have associated known genes (*LOC112445782*—28S ribosomal RNA, and *LOC100851913*—zinc finger protein 75D-like, respectively) and have high similarity to previously identified lncRNA (NONBTAT015718.2 and NONBTAT031715.1, respectively). The other lncRNA, TCONS_00141903, is located on chromosome 17 and is a non-coding isoform of gene *TXNRD2*. Two other DE lncRNA worth highlighting are TCONS_00051404 and TCONS_00051406. They were DE in muscle, presented high similarity with the previously identified NONBTAT026662.2, and are both isoforms of the same gene, *LOC104972733*, a ncRNA located on chromosome 6. The difference between these lncRNA is that TCONS_00051404 contains three instead of four exons, which gives it the potential to be a precursor of the bta-mir-11986 and also a binding site for seven miRNAs instead of two.

Among the 10 lncRNA DE in liver, four present high similarity (E-value < 10^−6^) with lncRNA found in the liver of taurine cattle and thought to be relevant to FE [21]. TCONS_00061987, TCONS_00128934, TCONS_00157869 and TCONS_00188391 correspond to the previously identified lncRNA, MSTRG.17590.2, MSTRG.9500.2, MSTRG.9500.8 and MSTRG.999.10, respectively (with identity between 83 and 90% and an E-value < 10^−13^).

### 3.4. Key lncRNA

**Figure 4:**
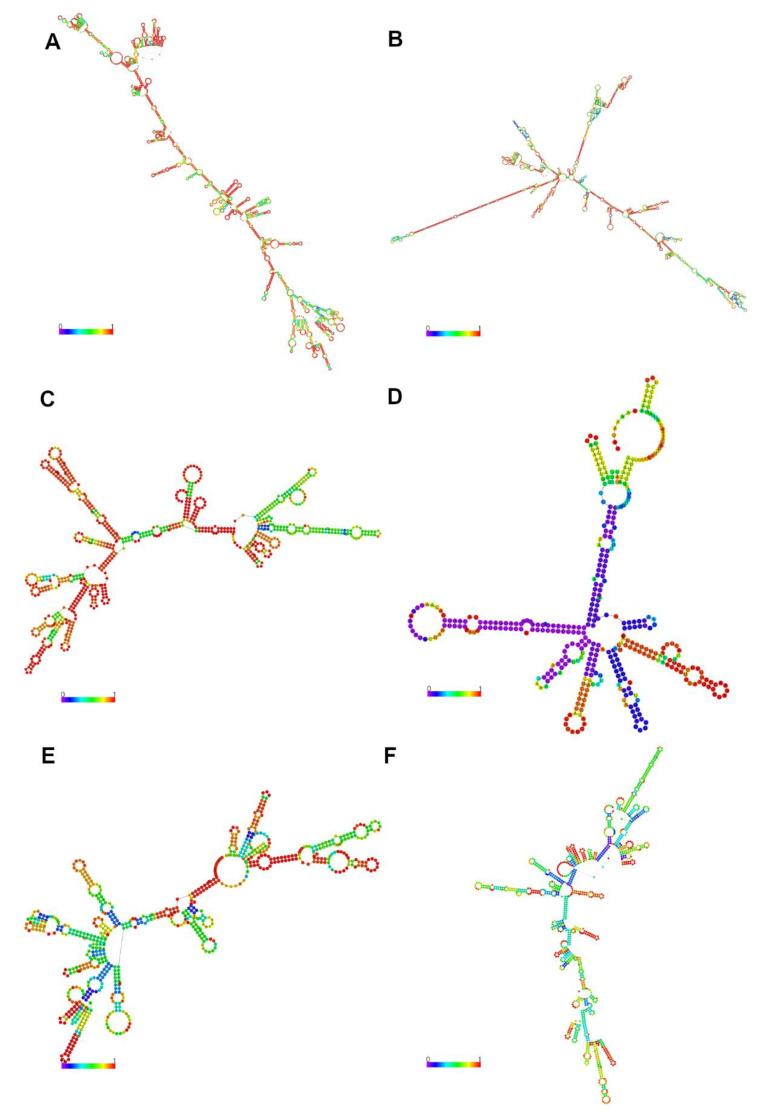
Minimum free energy structures encoding base-pair probabilities for lncRNA, color-coded from 0 (purple) to 1 (red). (**A**) TCONS_00051404—differentially expressed (DE) in muscle; (**B**) TCONS_00040537—DE and key regulator in adrenal gland; (**C**) TCONS_00111349—key regulator in liver; (**D**) TCONS_00106745—DE in liver and key regulator in adrenal gland; (**E**) TCONS_00222966—DE in hypothalamus and liver; (**F**) TCONS_00062811—DE in pituitary gland.

We were able to identify 71 key lncRNA with potential to be regulators of the expression of relevant known mRNA associated with FE in cattle (Table 2). Of those, 21 were identified in adrenal gland, eight in liver, 10 in muscle, 15 in hypothalamus and 17 in pituitary gland. No lncRNA were identified as key regulators in more than one tissue. Among the key lncRNA, two were also DE in the same tissue where their regulatory potential was identified: TCONS_00040537 in the adrenal gland and TCONS_00140963 in muscle. TCONS_00040537 (Figure 4B) contains a generic exonic which aligns with gene LOC112446864 (small nucleolar RNA SNORA44) and has high similarity with NONBTAT026662.2. TCONS_00140963 was originally an intergenic transcript but contains a generic exonic, which aligns with the predicted gene-NC_037344.1:67015837.67024675 and found no match in the NONCODE base.

Among the eight key lncRNA identified in liver, four have high similarity with lncRNA and are thought to be relevant to FE in taurine cattle [21]. TCONS_00056607, TCONS_00090296, TCONS_00111349 (Figure 4C) and TCONS_00190687 correspond to the previously identified lncRNA MSTRG.4390.1, MSTRG.8896.1, MSTRG.4330.3 and MSTRG.14754.11, respectively (with identity between 82 and 100% and E-value < 10^−9^).

### 3.5. Possible Functions of Relevant lncRNA for Feed Efficiency

**Figure 5:**
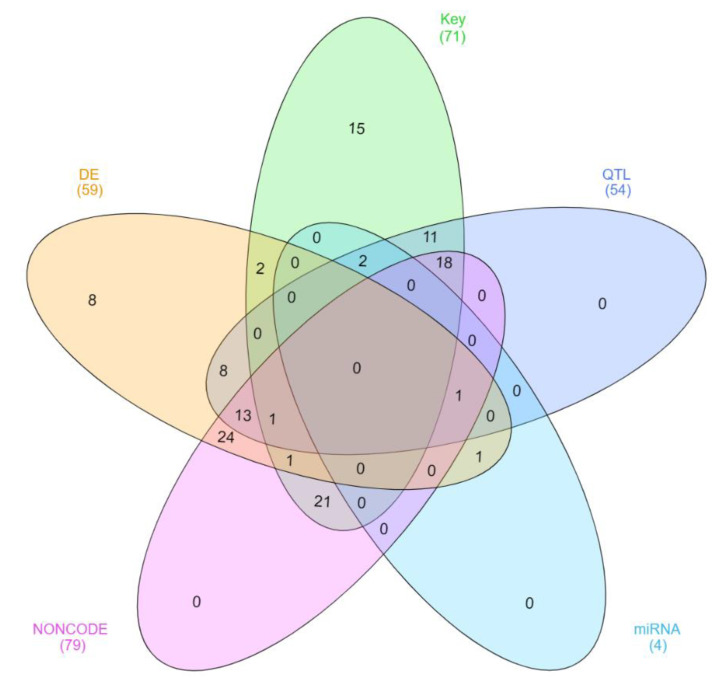
Number of DE (differentially expressed lncRNA) and Key (key regulator lncRNA) that are also quantitative trait loci (QTL) (overlap QTL for traits related to feed efficiency), miRNA (potential to be a miRNA precursor) and NONCODE (high similarity with previously described lncRNA).

Combining DE and key regulatory lncRNA, we identified 126 unique relevant transcripts which could be related to FE (considering four lncRNA were both DE and identified with regulatory potential). Several of these lncRNA, fall within genomic regions of QTL for traits related to FE, feed intake and fat deposition (Supplementary File S4, Figure 5). A total of 30 lncRNA are located in 56 QTL for RFI (the trait used to define the high and low FE groups). Among those, we highlight TCONS_00119451 and TCONS_00119463 for overlapping seven QTL for RFI (QTL:56461, QTL:20842, QTL:20843, QTL:20844, QTL:20845, QTL:20846, QTL:20847). TCONS_00119451 is a non-coding isoform of gene LOC104974057 (serine/arginine repetitive matrix protein 1-like) and a key lncRNA in muscle. TCONS_00119463 is a non-coding isoform of PEX2 and was DE in adrenal gland. Additionally, three lncRNA overlapped QTL for dry matter intake (DMI; TCONS_00032445, TCONS_00062811, TCONS_00149966) and two for feed conversion ratio (FCR; TCONS_00188391, TCONS_00190543).

One difference previously observed between high and low FE group animals was their fat deposition, both visceral and subcutaneous [27]. Of the 126 lncRNA identified as relevant to FE, 38 overlapped QTL for either rib fat thickness, rump fat thickness or kidney, pelvic and heart fat percentage. Of these, 20 were identified with regulatory potential, 17 were DE and one was both DE and had regulatory potential (TCONS_00040537). TCONS_00119451 and TCONS_00119463 overlap 11 QTL for fat deposition related traits. Conversely, two lncRNA (TCONS_00065862 and TCONS_00128697) overlap 39 QTL for lean meat yield, the proportion of lean meat on a carcass expressed as a percentage by weight. Both TCONS_00065862 and TCONS_00128697 are intergenic transcripts DE in hypothalamus. TCONS_00128697 corresponds to the previously identified NONBTAT027237.1.

Regarding the relationship between lncRNA and miRNA, four out of the 126 relevant lncRNA identified here showed potential to be miRNA precursors (Figure 5). Indeed, TCONS_00159585, TCONS_00159584 and TCONS_00170772 presented class code “o,” as they contained a generic exonic overlap with MIR22 and MIR154C. Although both TCONS_00159585 and TCONS_00159584 overlapped MIR22, TCONS_00159585 was identified as having regulatory potential in liver and TCONS_00159584 in adrenal gland. TCONS_00159584 corresponds to the predicted gene-NC_037346.1:22809076..22815378. TCONS_00170772 was DE in pituitary gland and overlapped MIR154C. Lastly, TCONS_00051404 (Figure 4A) is DE in muscle and is a potential precursor of bta-mir-11986. This lncRNA is also an isoform of the ncRNA LOC104972733 and has high similarity with NONBTAT026662.2. A large number (121 out of 126) of relevant lncRNA showed potential to act as miRNA sponges. Combined, the lncRNA had binding sites for 479 unique miRNA, while 230 of those miRNA had a binding site in two or more lncRNA. For instance, bta-miR-12059 had binding sites in nine lncRNA and bta-miR-2320-5p and eight bta-miR-149-3p both had binding sites in eight lncRNA. Nevertheless, no functional enrichment was found for miRNA targets.

### 3.6. lncRNA Co-Expression Networks

The network built for liver comprised 1447 mRNA and 40 lncRNA, with 18 of these lncRNA being either DE or having regulatory potential (Supplementary Figure S1). TCONS_00061987, a DE lncRNA that overlapped GLRA1 in the opposite strand and presented high similarity to NONBTAT029655.1, had the highest degree of connectivity, being directly connected to 567 mRNA and 10 lncRNA. Nevertheless, no significant functional enrichment was found among those mRNA. Another lncRNA of interest is TCONS_00106745 (Figure 4D), an intergenic lncRNA DE in liver and with regulatory potential in the adrenal gland. It was directly co-expressed in liver with 302 mRNA involved in the enrichment of cell adhesion molecules (FDR = 0.01) and complement and coagulation cascades (FDR = 0.01). In the adrenal gland, this same lncRNA was co-expressed with 210 mRNA, which are involved in valine, leucine and isoleucine degradation (FDR = 0.01) and drug metabolism (FDR = 0.01).

A further three lncRNA, TCONS_00188391, TCONS_00190687 and TCONS_00111349 presented functional enrichment of co-expressed mRNA in liver and with high similarity to lncRNA previously associated with FE in taurine cattle [21]. TCONS_00188391 is DE in liver, located in a QTL region for FCR and in the opposite strand to ACAA2, and has high similarity to the previously described NONBTAT026662.2. It is co-expressed with 95 genes enriched for regulation of leukocyte activation (FDR = 0.05), eicosanoid transport and secretion (FDR = 0.05), fatty acid derivative transport (FDR = 0.05) and lymphocyte activation involved in immune response (FDR = 0.097). TCONS_00190687 is an intergenic lncRNA with regulatory potential and has high similarity to NONBTAT031971.1. This lncRNA was co-expressed with 385 genes involved in steroid hormone biosynthesis (FDR = 0.078) and complement and coagulation cascades (FDR = 0.078). Finally, key lncRNA TCONS_00111349 (Figure 4C) is an isoform of the ncRNA LOC100847759 and has high similarity to NONBTAT027933.1. This lncRNA is co-expressed with 148 mRNA, including the key regulator of FE NR2F6, which are involved in lipid homeostasis (FDR = 0.057) and cholesterol metabolism (FDR = 0.084).

The adrenal gland network included 1875 mRNA and 64 lncRNA, with 33 of these lncRNA being either DE or having regulatory potential (Supplementary Figure S2). Within the network, TCONS_00180358 presented the highest degree of connectivity, being directly co-expressed with 495 mRNA and seven lncRNA. TCONS_00180358 was DE and overlapped LOC100847326 (an uncharacterized ncRNA) in the opposite strand, but the mRNA this lncRNA was co-expressed with displayed no significant functional enrichment. A lncRNA worth highlighting is TCONS_00040537 (Figure 4B) which was both DE and had regulatory potential. This lncRNA was directly co-expressed with 94 mRNA, involved in the immune response (FDR = 0.078) and regulation of cell substrate adhesion (FDR = 0.078).

The hypothalamus network presented 1,424 mRNA and 57 lncRNA, with 24 of these lncRNA being either DE or having regulatory potential (Supplementary Figure S3). The lncRNA with the highest degree of connectivity was TCONS_00139694, which is DE in the hypothalamus and overlaps SCARB1 in the opposite strand. This transcript corresponded to the predicted gene-NC_037344.1:50924544..50931802 and had high similarity to NONBTAT031990.1. This lncRNA was directly co-expressed with 535 mRNA but these mRNA displayed no significant functional enrichment. In contrast, TCONS_00222966 (Figure 4E), which was DE in both the hypothalamus and liver, was directly co-expressed with 37 mRNA involved in cellular calcium ion homeostasis (FDR = 0.034), tachykinin receptor signaling (FDR = 0.046) and sensory perception (FDR = 0.05). TCONS_00141903 was also DE in all tissues of the HPA axis and, in the hypothalamus, it was co-expressed with 34 mRNA involved in cellular responses to organic substances (FDR = 0.093).

The pituitary gland network included 1325 mRNA and 59 lncRNA, with 34 of these lncRNA being either DE or having regulatory potential (Supplementary Figure S4). The lncRNA with the highest degree of connectivity was TCONS_00062811 (Figure 4F), being co-expressed with 156 mRNA. This lncRNA was DE in pituitary gland and is located in a QTL region for DMI and overlaps GFPT2 in the opposite strand. This lncRNA is co-expressed with mRNA involved in negative regulation of protein phosphorylation (FDR = 0.067). TCONS_00141903, already mentioned for being DE in all tissues of the HPA axis and, in the pituitary gland, it was co-expressed with 34 mRNA involved in arginine and proline metabolism (FDR = 0.052).

Finally, the network build for muscle was comprised of 1029 mRNA and 38 lncRNA, with 23 of these lncRNA being either DE or having regulatory potential (Supplementary Figure S5). TCONS_00011978 is a lncRNA with regulatory potential and displayed the highest degree of connectivity being directly connected to 190 mRNA. It contains a generic exonic overlap with CCNYL1, high similarity to NONBTAT026052.2 and is co-expressed with mRNA involved in arginine and proline metabolism (FDR = 0.09). Two additional lncRNA, that are isoforms of the same gene, presented different behaviors in the network. While TCONS_00051406 was co-expressed with 64 mRNA with no functional enrichment, TCONS_00051404 (Figure 4A) was co-expressed with 165 mRNA that were highly enriched for ribosome (FDR = 0.009).

## 4. Discussion

The growing number of next-generation sequencing data and the development of computational biology have brought the regulatory role of lncRNA in biological processes to light, which may offer a deeper understanding of the phenotypic variation of complex traits in farm animals [2,52]. In this study, we used transcriptomic data from 18 Nellore bulls, to identify lncRNA expressed in five tissues (hypothalamus, pituitary gland, adrenal gland, muscle and liver) of indicine cattle. The functional characterization of lncRNA is difficult since there is low conservation between species and their function is sometimes determined by their three-dimensional structure [19]. Nevertheless, by contrasting high and low FE animals, we were able to highlight 126 lncRNA relevant to FE and infer their function by exploring their relationship with miRNA, co-expression with mRNA relevant to FE and overlap with QTL regions for correlated phenotypes. Some lncRNA associated with FE were of particular interest as their relevance to FE was indicated by several different approaches and will be further discussed below.

Few studies have attempted to catalogue lncRNA in multiple bovine tissues [18,53], with one such study having a specific focus on FE [20]. However, to date, all studies have been conducted using taurine breeds. Given the regulatory role of lncRNA and their apparent lack of conservation between species [2], it is expected that important differences may exist between indicine and taurine cattle, especially regarding adaptability and divergent selection [22]. Therefore, our first goal was to identify novel lncRNA in Nellore cattle using successive filtering of novel transcripts. The filter that excluded most transcripts was the requirement for the presence of ORFs. Although the presence of a START and STOP codon does not guarantee the translation of a functional protein, this possibility must be ruled out when investigating lncRNA [33]. The CPC2 filter was the final filter used and was in place to ensure the other filters were efficient at identifying lncRNA. It excluded only seven transcripts, giving us confidence that our method was accurate in identifying lncRNA. A total of 55% of the transcripts identified as lncRNA showed homology with the bovine NONCODE database. This indicates on the one hand that the methodology used here was efficient at identifying lncRNA, while on the other hand, this highlights deficiencies in the annotation of these molecules, particularly considering differences between taurine and indicine species.

Regarding classification of the lncRNA, the most represented class code was “j” (38%, new isoforms of known genes), followed by “u” (23%, intergenic transcripts), also known as long intergenic non-coding RNA (lincRNA). These proportions change dramatically when we add to the reference transcripts that are predicted to exist based on other genomes and search algorithms. Non-coding isoforms of reference genes increase to 50%, while lincRNA decline to only 8%, with the appearance of other class codes associated with overlaps with reference transcripts, such as “i,” “c” and “s.” A total of 3% of all lncRNA identified in this study completely overlap predicted transcripts. Based on these findings we can argue that the better the genome is annotated, the less lncRNA is classified as lincRNA. Although different studies in cattle and other production species indicate the presence of most lncRNA in intergenic regions [18,53], both the results from the current study and the study by [21], using the new bovine genome (ARS-UCD1.2), suggest only a small proportion (~15%) of lncRNA are characterized as lincRNA. It is evident that much of mammalian genomes remain unexplored and has been referred to as “dark genome” [54]. Future utilization of the new bovine genome will help to improve our understanding of these molecules.

Of the total identified lncRNA, 888 were expressed at sufficient levels between the tissues to be tested for differential expression and of these, only 12% were common to the five tissues reflecting the tissue-specificity expected for these RNA [2,3]. The tissues with the highest number of lncRNA in common are those of the HPA axis, with 76 transcripts expressed in hypothalamus, pituitary gland and adrenal gland. This finding may be related to the regulation of the endocrine function exercised in common by these tissues in which lncRNA could be involved. This same pattern was observed for DE genes with only three out of the 59 lncRNA being DE in more than one tissue, mostly from the HPA axis. Regarding lncRNA with regulatory potential, no lncRNA were identified in more than one tissue, once more reflecting the tissue specificity of these molecules and also their unique regulatory function. Similar to transcription factors, lncRNA present lower transcript abundance compared with mRNAs [5,43] and subtle changes between conditions, although not captured by differential expression, could reflect an important regulatory role for these molecules [20]. This is evidenced here by the higher number of lncRNA identified as having regulatory potential (n = 71) compared to those identified as being DE (*n* = 59).

The liver is the most studied organ in the context of FE due to the variety of immunological and metabolic functions it performs. At the molecular level, the majority of studies point to genes and pathways involved in oxidative stress, lipid metabolism, inflammation and/or immune response being important for liver function [13,14,15,27,55,56]. In our study, four DE and four lncRNA with regulatory potential were identified that had high similarity with lncRNA identified as being relevant to FE in the liver of taurine cattle [21]. Among those, TCONS_00188391, TCONS_00111349 and TCONS_00190687 were co-expressed with mRNA involved in lymphocyte and leukocyte activation, complement and coagulation cascades, lipid homeostasis, cholesterol metabolism and steroid hormone biosynthesis. Indeed, inflammation-associated processes and heightened immune responses have been reported in the liver of low FE cattle [14,56,57,58]. Moreover, both altered lipid metabolism and steroid hormone biosynthesis in liver have also been associated with FE [13,27,55,59], with low FE animals having higher fat deposition and higher cholesterol levels [60,61,62,63].

**Figure 6:**
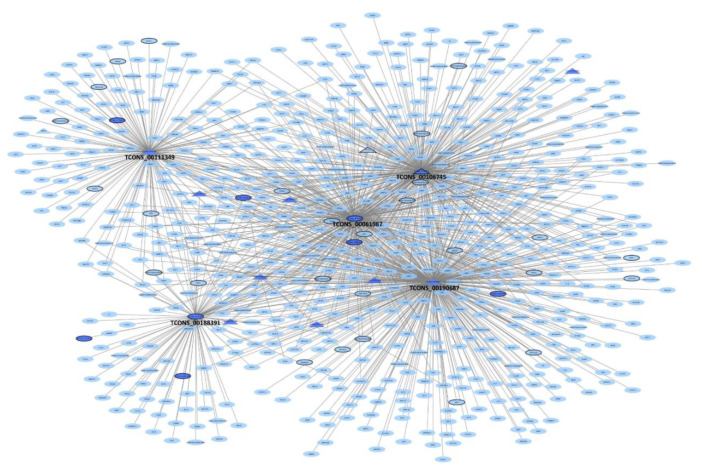
Liver subnetwork comprised of the first neighbors of TCONS_00188391, TCONS_00111349, TCONS_00190687 and TCONS_00106745. The light color indicates mRNA and the dark color lncRNA; differentially expressed (DE) mRNA and lncRNA are indicated by the black border; key lncRNA and mRNA are indicated as a triangle. DE and key mRNA correspond to results from Alexandre et al. (2019 25).

The exact function of lncRNA is difficult to predict in silico; however, by overlapping different sources of information one can predict the function of molecules with some level of accuracy. TCONS_00106745, an intergenic lncRNA shown to be DE in liver and to have regulatory potential in adrenal gland. This lncRNA had no overlap with any previously identified lncRNA but was predicted to be involved in cell adhesion and complement and coagulation cascades, processes related to inflammation [64]. Therefore, this lncRNA shows potential to be specifically associated with FE in *B. indicus*. Interestingly, when we create a subnetwork only with the first neighbors of the four discussed lncRNA (Figure 6), TCONS_00061987, it appears in the center of the network. Indeed, TCONS_00061987 is the lncRNA with the highest degree of connectivity in the overall liver network and has high similarity with a lncRNA thought to be associated with FE in taurine cattle.

The muscle has also been investigated in the context of FE, particularly at the molecular level [16,17,20,65,66,67]. It is common for beef producers to simultaneously target improved FE, increased muscling and higher marbling in their animals [68]. In the current study, TCONS_00051404 is of particular interest as this lncRNA is DE in muscle, has overlapping QTL for RFI and visceral fat, is a possible precursor of bta-mir-11986 and is an isoform of a known ncRNA. This lncRNA is co-expressed with mRNA involved in ribosome biogenesis and which have been reported to influence muscling in *B. indicus* cattle with divergent FE [17,65]. It has been hypothesized that ribosome biogenesis may play an important role in the regulation of skeletal muscle growth and increased expression of ribosomal genes, which have positive implications for FE [69]. The efficiency of translation affects protein synthesis rate, which in turn is directly impacted by the number of ribosomes providing a possible mechanism for this effect [70,71]. Furthermore, both in pigs and cattle, increased FE is associated with stimulation of muscle development and growth, which seems to be regulated by the *TGFB1* signaling pathway [25,66,72].

A regulatory role of lncRNA in myogenesis and muscle cell differentiation has been reported in different species [73,74,75,76,77]. Nolte et al. (2019) identified a lncRNA associated with FE in *Bos taurus* cattle (using UMD3.1 genome), whose expression was negatively correlated with L-arginine plasma levels and was hypothesized to play an inhibitory role in metabolic efficiency of dairy cows. Concordantly, our study identified two lncRNA worth highlighting in this context. TCONS_00011978 was identified as having regulatory potential in muscle and had the highest degree of connectivity within muscle network, reaffirming its potential as a central regulator. This lncRNA was co-expressed with mRNA involved in arginine and proline metabolism. In indicine cattle, arginine and proline metabolism have been associated with divergent selection for growth [78]. Another lncRNA with regulatory potential in muscle was TCONS_00119451, a non-coding isoform of gene *LOC104974057* known as serine/arginine repetitive matrix protein 1-like. This lncRNA overlapped seven QTL for RFI and 11 QTL for fat deposition related traits and is also thought to be related to FE [60,61,62,63,79]. Amino acids are the building blocks of proteins and intermediates in metabolism and are therefore intrinsically involved in muscle growth and development [78,80]. Finally, TCONS_00140963 was both DE and had regulatory potential in muscle, but no clear evidence of its role in FE could be elucidated making it a candidate molecule for further studies.

The role of the HPA axis in FE is largely unknown, although studies at molecular level suggest the HPA axis may play a role in FE [25,81,82,83]. In cattle, the HPA axis has been implicated in the relationship between FE and temperament [84]. While most other studies have focused on understanding the influence of leptin on FE, due to its role in regulating body weight, feed intake and energy expenditure [79,85,86,87,88]. Nevertheless, studies trying to elucidate the complex regulatory mechanisms by which the hypothalamus regulates FE have yielded variable results [89,90], indicating the need for more studies in this area. In the current study, the lncRNA TCONS_00222966 was DE in both hypothalamus and liver. Additionally, its co-expressed genes play a role in cellular calcium ion homeostasis, tachykinin receptor signaling and sensory perception. The stimulation of tachykinin receptors leads to an elevation of intracellular calcium levels, which modulates the activity and release of other neurotransmitters such as dopamine and norepinephrine [91]. The role of tachykinins in the central nervous system is not fully understood but may be related to somatic and visceral sensory integration and be important in learning, memory and behavioral responses [91]. Indeed, dopamine is a key neurotransmitter modulating the rewarding effects of food, leading to food-seeking behaviors [92,93,94]. Based on previously reported links between feeding behavior pathways in hypothalamus and FE [83], it is tempting to speculate a possible role of TCONS_00222966 in FE.

The lncRNA with the highest degree of connectivity in hypothalamus was TCONS_00139694, which is DE and overlaps *SCARB1* in the opposite strand. SCARB1 is a key component in the reverse cholesterol transport pathway and thus may play an important role in lipid metabolism [95]. Two DE lincRNA in hypothalamus (TCONS_00065862 and TCONS_00128697) overlapped 39 QTL for lean meat yield. Moreover, TCONS_00119463, DE in the adrenal gland, overlapped seven QTL for RFI and 11 QTL for fat deposition related traits. TCONS_00040537, which was DE, had regulatory potential in the adrenal gland, overlapped QTLs for fat deposition-related traits and is co-expressed with mRNA involved in immune response and regulation of cell-substrate adhesion. Another lncRNA worth highlighting is TCONS_00141903, which was DE in the hypothalamus, pituitary gland and adrenal gland. It is a non-coding isoform of *TXNRD2*, a gene involved in the control of reactive oxygen species levels, regulation of mitochondrial redox homeostasis and is thought to play a role in redox-regulated cell signaling. The functional relationship between lncRNA might inform predictions of their functions [21]. It is known that the HPA axis plays an important role in the control of body weight, ingestion and fat metabolism and that, in humans, pathologies caused by fat accumulation can lead to inflammatory responses in many tissues, including those in the HPA axis [96,97]. Currently, little is known about these mechanisms in cattle and how they relate to FE; however, our results strongly support an important role for lncRNA in regulating FE in indicine cattle.

## 5. Conclusions

In this study, we were able to identify new lncRNA in five tissues of Nellore cattle, which are predicted to be involved in the regulation of FE of indicine cattle. To explore similarities and differences in the role that lncRNA plays in regulating FE in taurine and indicine cattle will require investigation of data from both sub-species using the same pipeline and reference genome. Nevertheless, we can be confident from the results of the current study that there are lncRNA specifically involved in FE regulation of indicine cattle. Therefore, this study contributes to expanding the catalogue of lncRNA candidates that are predicted to play a role in regulating FE and will help to elucidate the molecular mechanisms involved in the regulation of this complex trait. The lncRNA highlighted here are candidates for further studies in animal selection and animal management.

## Figures and Tables

**Table 1:** Differentially expressed (DE) long non-coding RNA.

Tissue	DE IncRNA
Adrenal gland	TCONS_00223090, TCONS_00141903, TCONS_00214308, TCONS_00040537, TCONS_00119463, TCONS_00093659, TCONS_00180358, TCONS_00072894, TCONS_00034840, TCONS_00164459, TCONS_00027608, TCONS_00015370, TCONS_00127543
Hypothalamus	TCONS_00222966, TCONS_00128697, TCONS_00016951, TCONS_00065862, TCONS_00106598, TCONS_00157676, TCONS_00083779, TCONS_00139694, TCONS_00141903
Liver	TCONS_00106745, TCONS_00130767, TCONS_00061987, TCONS_00025987, TCONS_00128934, TCONS_00157869, TCONS_00222578, TCONS_00222972, TCONS_00188391, TCONS_00222966
Muscle	TCONS_00140963, TCONS_00223154, TCONS_00128551, TCONS_00032445, TCONS_00095545, TCONS_00000271, TCONS_00141506, TCONS_00051404, TCONS_00120014, TCONS_00033623, TCONS_00203516, TCONS_00051406, TCONS_00167041, TCONS_00190543
Pituitary gland	TCONS_00116172, TCONS_00032383, TCONS_00105367, TCONS_00077897, TCONS_00157315, TCONS_00202013, TCONS_00062811, TCONS_00009194, TCONS_00131281, TCONS_00150705, TCONS_00170772, TCONS_00116008, TCONS_00168127, TCONS_00188529, TCONS_00059814, TCONS_00223090, TCONS_00141903

**Table 2:** Key long non-coding RNA according to the Regulatory Impact Factor algorithm.

Tissue	Key IncRNA
Adrenal gland	TCONS_00106745, TCONS_00040537, TCONS_00006522, TCONS_00013774, TCONS_00022218, TCONS_00048225, TCONS_00064059, TCONS_00065193, TCONS_00065195, TCONS_00083522, TCONS_00088984, TCONS_00126728, TCONS_00154980, TCONS_00159584, TCONS_00171940, TCONS_00178323, TCONS_00182439, TCONS_00186763, TCONS_00193324, TCONS_00201789, TCONS_00219008
Hypothalamus	TCONS_00214308, TCONS_00018896, TCONS_00028218, TCONS_00028219, TCONS_00033000, TCONS_00061315, TCONS_00068546, TCONS_00153695, TCONS_00157240, TCONS_00157945, TCONS_00164540, TCONS_00169707, TCONS_00176859, TCONS_00187047, TCONS_00198904
Liver	TCONS_00056607, TCONS_00079733, TCONS_00090296, TCONS_00096860, TCONS_00111349, TCONS_00159585, TCONS_00185398, TCONS_00190687
Muscle	TCONS_00140963, TCONS_00011978, TCONS_00028495, TCONS_00064224, TCONS_00103343, TCONS_00116181, TCONS_00119451, TCONS_00122105, TCONS_00135035, TCONS_00171719
Pituitary gland	TCONS_00006521, TCONS_00012621, TCONS_00018857, TCONS_00024003, TCONS_00029744, TCONS_00045668, TCONS_00053912, TCONS_00056694, TCONS_00116405, TCONS_00140488, TCONS_00142880, TCONS_00149966, TCONS_00166200, TCONS_00184540, TCONS_00184673, TCONS_00202748, TCONS_00222510

## Supplementary Materials

The following are available online at https://www.mdpi.com/2073-4425/11/9/997/s1, Supplementary File S1: Annotation of new lncRNA, Supplementary File S2: Table of attributes for new lncRNA, Supplementary File S3: Differential expression results; Supplementary File S4: lncRNA overlapping QTL regions for traits associated with feed efficiency; Supplementary Figure S1: Liver network *, Supplementary Figure S2: Adrenal gland network *; Supplementary Figure S3: hypothalamus network *, Supplementary Figure S4: Pituitary gland network *, Supplementary Figure S5: Muscle network *. * In the networks, light color indicates mRNA and the dark color indicates lncRNA; differentially expressed (DE) mRNA and lncRNA are indicated by the black border; key lncRNA and mRNA are indicated as a triangle. DE and key mRNA correspond to results from Alexandre et al. (2019).
