# Supplementary figures and images for: Exploring the Regulatory Potential of Long Non-Coding RNA in Feed Efficiency of Indicine Cattle
Source: Genes (Basel). 2020 Aug 25;11(9):997. doi: 10.3390/genes11090997 (PMC7565090; doi:10.3390/genes11090997)

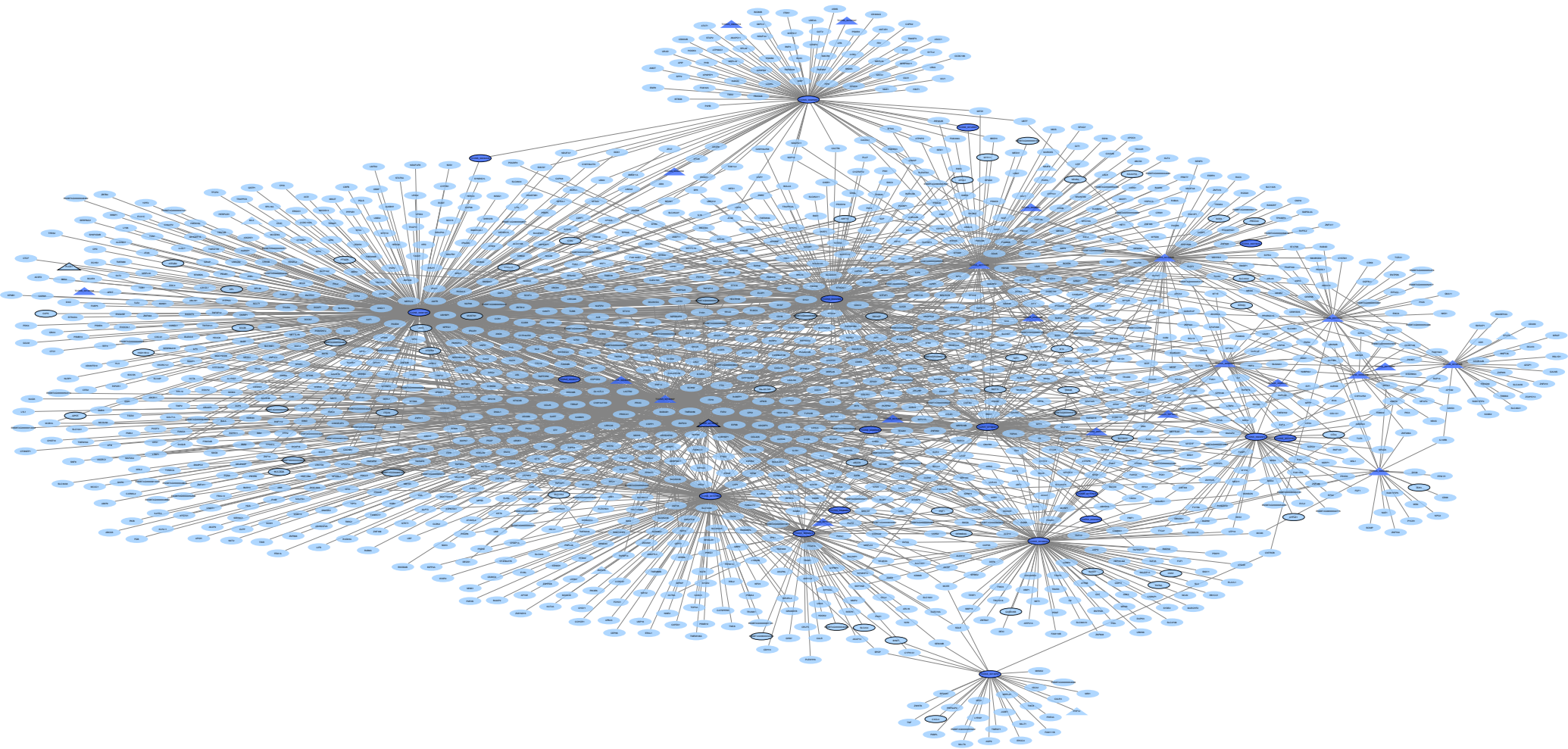

Supplement: Supplementary file 1 [file genes-11-00997-s001.zip › SupplementaryFigure1_LiverNetwork.pdf]

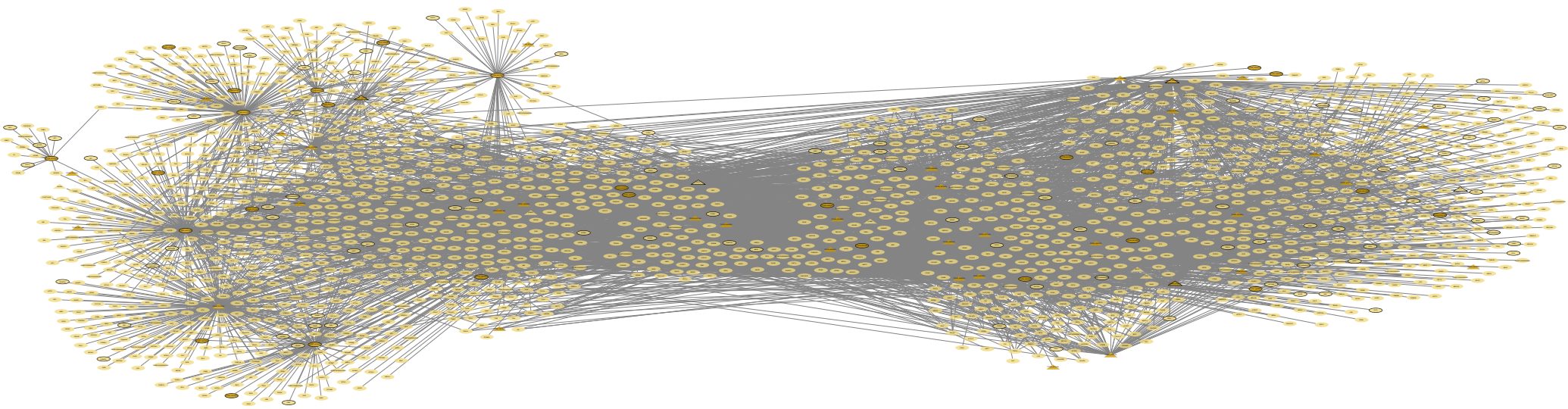

Supplement: Supplementary file 1 [file genes-11-00997-s001.zip › SupplementaryFigure2_AdrenalNetwork.pdf]

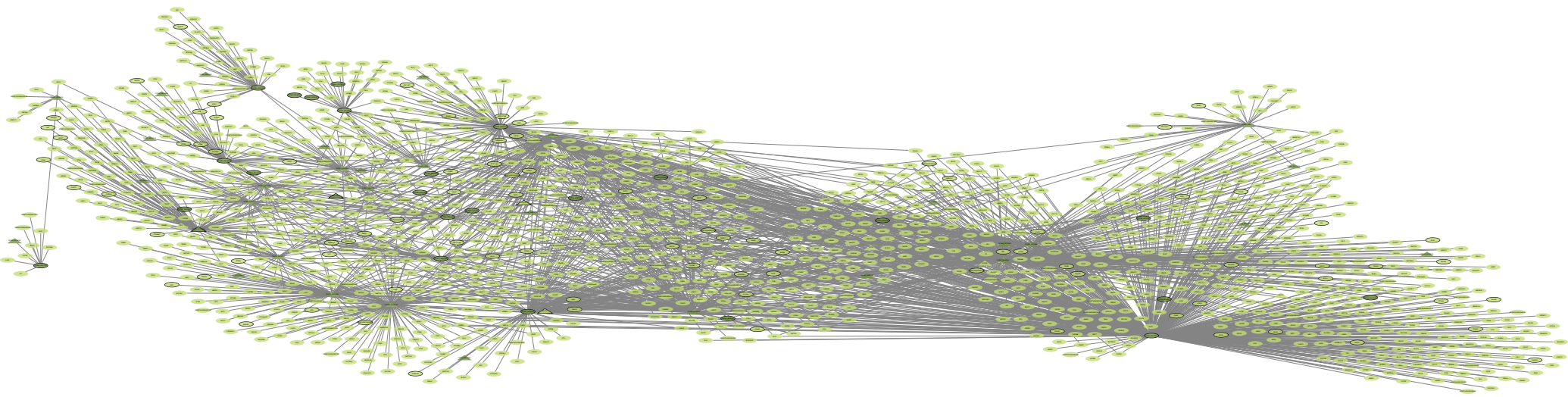

Supplement: Supplementary file 1 [file genes-11-00997-s001.zip › SupplementaryFigure3_HypothalamusNetwork.pdf]

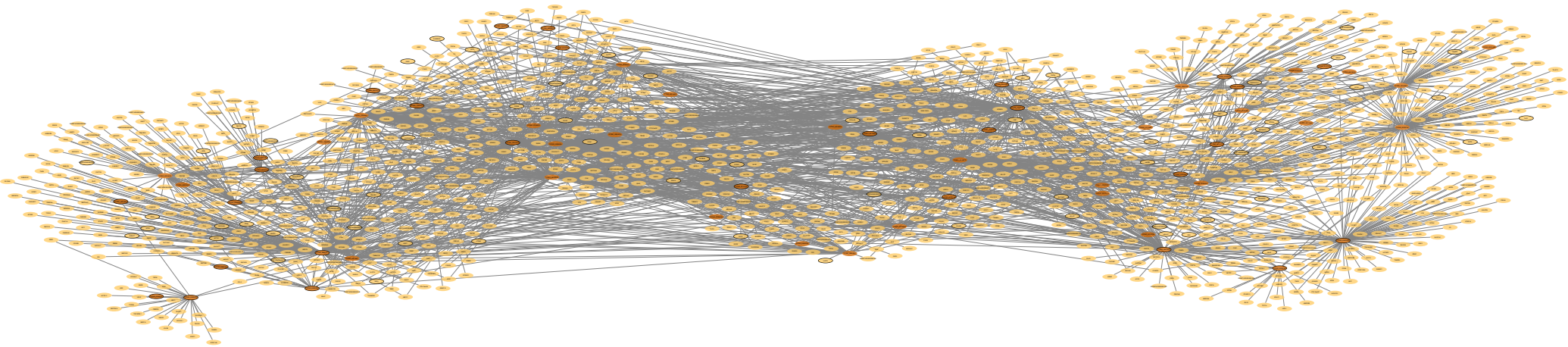

Supplement: Supplementary file 1 [file genes-11-00997-s001.zip › SupplementaryFigure4_PituitaryNetwork.pdf]

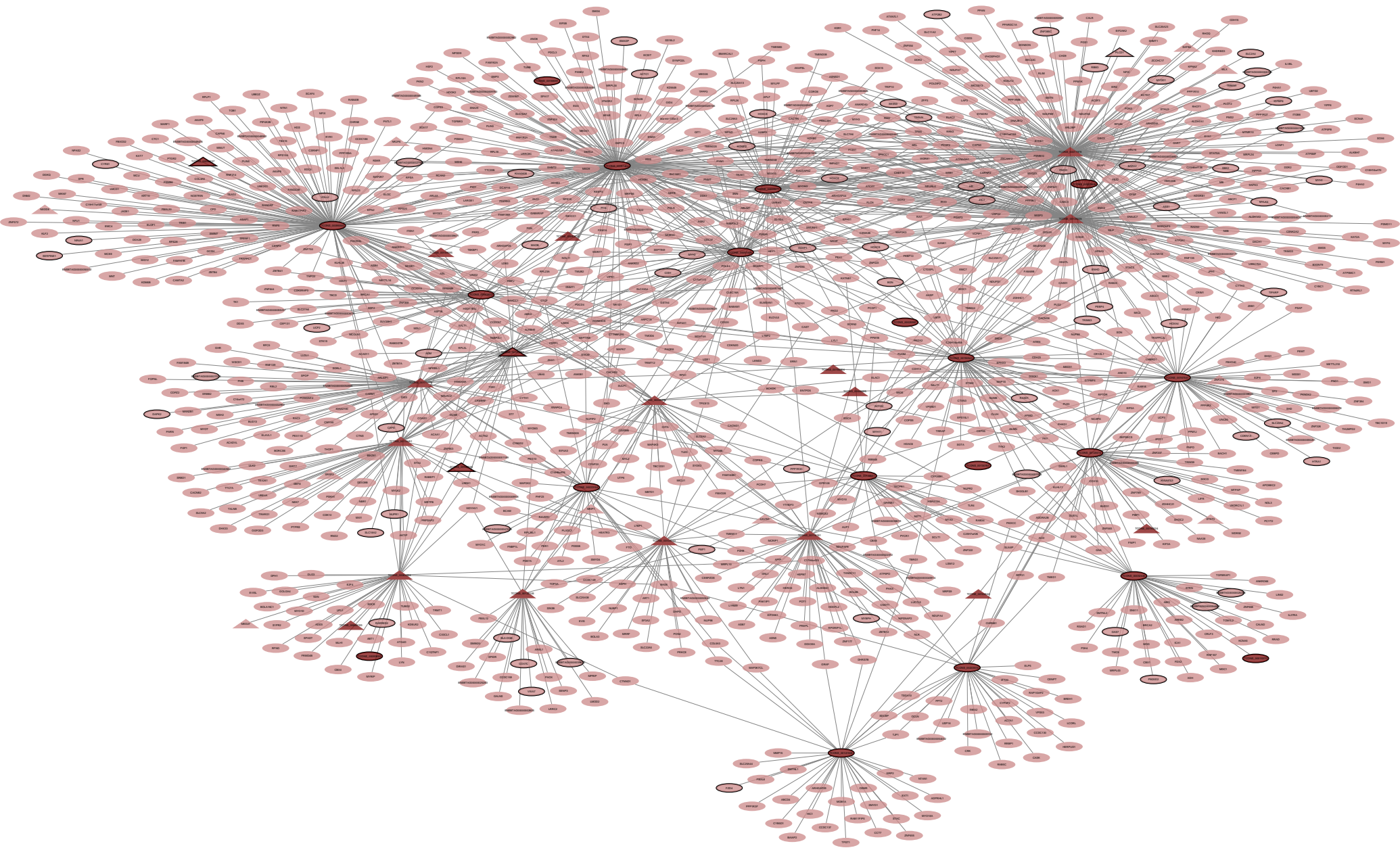

Supplement: Supplementary file 1 [file genes-11-00997-s001.zip › SupplementaryFigure5_MuscleNetwork.pdf]
